# Impact of aldosterone deficiency on the development of diuretic resistance in mice

**DOI:** 10.1007/s00424-025-03082-8

**Published:** 2025-04-12

**Authors:** Daniel Essigke, M. Zaher Kalo, Andrea Janessa, Bernhard N. Bohnert, Xiaqing Li, Andreas L. Birkenfeld, Ferruh Artunc

**Affiliations:** 1 Department of Internal Medicine, Division of Diabetology, Endocrinology and Nephrology, University Hospital Tübingen, Otfried-Mueller-Str.10, 72076 Tübingen, Germany; 2 Institute of Diabetes Research and Metabolic Diseases (IDM), Helmholtz Center Munich, University Tübingen, Tübingen, Germany; 3 German Center for Diabetes Research (DZD), University Tübingen, Tübingen, Germany

**Keywords:** (MeSH): Aldosterone, Aldosterone synthase, Diuretics, Furosemide, Hydrochlorothiazide, Triamterene

## Abstract

The effect of diuretics can be limited by stimulation of counter-regulatory mechanisms, eventually leading to diuretic resistance. It is thought that the mineralocorticoid aldosterone might contribute to the development of diuretic resistance. To test this, we challenged genetically modified mice with or without a deletion of the gene coding for the aldosterone synthase (AS) with furosemide, hydrochlorothiazide (HCT) and triamterene. Urinary excretion was studied in metabolic cages; kidneys were studied for expression of sodium transporters. In both genotypes, a 4-day treatment with HCT via drinking water (400 mg/l) induced a similar natriuresis and modest loss of body weight < 10%. In contrast, furosemide (125 mg/l) and triamterene (200 mg/l) via drinking water stimulated a significantly higher natriuresis and body weight loss in *AS*^−/−^ mice and in addition, triamterene caused massive hyperkalemia > 9 mM and acidosis (pH < 7.0). In *AS*^+*/*+^ mice, plasma aldosterone concentration tended to increase under furosemide and HCT administration, while triamterene induced a robust ~ sixfold increase. In the kidney, apical targeting and proteolytic activation of the epithelial sodium channel ENaC were stimulated in *AS*^+*/*+^ mice under triamterene treatment, an effect that was diminished in *AS*^−*/*−^ mice. In conclusion, aldosterone is essentially involved in the development of diuretic resistance to ENaC blockade by triamterene and to a lesser extent to furosemide. In contrast, resistance to HCT was independent of aldosterone.

**Supplementary Information:**

The online version contains supplementary material available at 10.1007/s00424-025-03082-8.

## Introduction

The development of edema in heart and kidney disease is caused by renal sodium and water retention, which occurs via various mechanisms, including the activation of the renin–angiotensin–aldosterone system (RAAS) or proteinuria/proteasuria [1, 2]. In addition to edema of the lower extremities, effusions frequently occur in the pleura, peritoneum, or pericardium, which markedly elevates the morbidity and mortality [3]. In clinical practice, fluid overload is a common cause of hospitalization in these patients, making diuretics essential for treatment and relief of edema. Diuretics are classified according to the site of their action and clinically relevant diuretics include loop diuretics (e.g., furosemide) acting on the Na^+^-K^+^-Cl^−^- cotransporter (NKCC2), thiazides (e.g., hydrochlorothiazide, HCT) acting on the sodium-chloride symporter (or Na^+^-Cl^−^-cotransporter NCC) and potassium-sparing diuretics (e.g., triamterene) acting on the epithelial sodium channel ENaC, each by blocking the channel’s pore. In this context, blockers of the mineralocorticoid receptor (MR) do not exert a direct diuretic effect but reduce ENaC-mediated sodium transport. Repeated administration of diuretics invariably provokes counter-regulation, which limits their natriuretic effects and as a consequence leads to a state of diuretic resistance [4]. The precise mechanisms underlying diuretic resistance remain unclear. It is postulated that, on the one hand, the renal sodium transporters are upregulated, mainly at their site of action [5]. On the other hand, there is likely a stimulation of the renin-angiotensin system (RAS) with the development of hyperaldosteronism [6]. It is noteworthy that concomitant administration of an angiotensin-converting enzyme (ACE) inhibitor did not alter the sodium and potassium balance during diuretic therapy [7].

Aldosterone plays a crucial role in sodium handling due to its effects on sodium reabsorption in the distal tubule, particularly in the aldosterone-sensitive distal nephron. Aldosterone is synthesized from cholesterol via multiple steps by the aldosterone synthase (AS, *Cyp11b2*) in adrenocortical cells [8]. In the distal tubule, aldosterone regulates the expression of the NCC and the ENaC [9–11]. In the late distal tubule aldosterone interacts with the principal cells by binding to the mineralocorticoid receptor, resulting in the upregulation of the transcription of the alpha subunit of ENaC [12, 13]. Furthermore, it has been demonstrated that serum-glucocorticoid regulated kinase 1 (SGK1) can be activated by aldosterone, which subsequently contributes to an increase in transcription, membrane abundance via modulation of Nedd4-2-mediated ubiquitination activity and the open probability of ENaC itself [14, 15].

A valuable model to study the effects of aldosterone in vivo are mice lacking the gene encoding for aldosterone synthase (*AS*^−/−^) [16, 17]. In contrast to adrenalectomized mice or mice lacking the mineralocorticoid receptor [18, 19], *AS*^−/−^ mice are compensated under a control diet and do not require supplementation of saline. In this study, we used *AS*^−/−^ mice to investigate the response to acute and chronic treatment with diuretics with regard to the development of diuretic resistance. We found that the absence of aldosterone interferes with the development of diuretic resistance to NKCC2 and ENaC blockade, but not to HCT.

## Materials and methods

### Mouse studies

Experiments were performed on 3- to 6-month-old genetically modified knock out mice carrying a mutation of the gene encoding the aldosterone synthase (*Cyp11b2*^*tm1Hsk*^), as described by Lee et al. [16]. The mice were a kind gift from the University of Zurich, Switzerland on a 129S6/SvEvTac background and backcrossed onto a 129S1/SvImJ background for ten generations. Genotyping was done using PCR. Mice were kept on a 12:12-h light–dark cycle and fed a standard chow (ssniff, V1534, Soest, Germany) with tap water ad libitum.

In healthy *AS*^+*/*+^ and *AS*^−/−^-mice, renal sodium handling was studied in metabolic cages. Mice were kept for 2 days on a control diet (C1000, sodium and potassium content 110 µmol/g and 178 µmol/g, respectively, Altromin, Lage, Germany). To investigate the responses to diuretics, mice were maintained in metabolic cages and treated for 4 days with the ENaC inhibitor triamterene (200 mg/L in the drinking water, pH 3), the NKCC2 inhibitor furosemide (125 mg/L in the drinking water) or the NCC inhibitor hydrochlorothiazide (400 mg/L in the drinking water, pH 4) as described in previous studies [20–22]. An end point was defined by loss of more than 20% of body weight compared to baseline or deterioration, necessitating termination of the experiment. Acute diuretic response was studied by bolus administration of 10 µg/g i.p. of each diuretic and subsequent collection of urine for 6 h without access to water and food.

All mouse experiments were conducted according to the National Institutes of Health Guide for the Care and Use of Laboratory Animals and the German law for the welfare of animals, and were approved by local authorities (Regierungspraesidium Tuebingen).

### Laboratory measurements

Urinary creatinine was measured with a colorimetric Jaffé assay (Labor + Technik, Berlin, Germany), urinary sodium and potassium concentration as well as fecal sodium content (after dissolution in nitric acid) with flame photometry (Eppendorf EFUX 5057, Hamburg, Germany). 24 h urinary sodium and potassium excretion was normalized to body weight. Plasma urea was measured enzymatically using a colorimetric assay (Labor + Technik, Berlin, Germany), plasma aldosterone was measured using an ELISA kit (IBL, Hamburg, Germany). Plasma sodium and potassium were measured using an IL GEM® Premier 3000 blood gas analyzer (Instrumentation Laboratory, Munich, Germany).

### Western blot from kidney tissue of mice

Western blot analysis of ENaC subunits as well as NCC, NKCC2, and ROMK was performed from a membrane protein preparation of kidney cortex collected under control condition or continuous diuretic treatment. Half of the frozen kidney per mouse was sliced, and the cortex was dissected using a scalpel. Homogenization was performed using a Dounce homogenisator in 1 mL lysis buffer containing 250 mM sucrose, 10 mM triethanolamine HCl, 1.6 mM ethanolamine and 0.5 EDTA at pH 7.4 (all Sigma) [23]. During all preparation steps, aprotinin (40 µg/mL) and a protease inhibitor cocktail (final concentration 0.1 × stock; mini-complete, Roche) was present to avoid ENaC cleavage in vitro. Homogenates were centrifuged at 1000* g* for removal of the nuclei. Subsequently, the supernatant was centrifuged at 20,000* g* for 30 min at 4 °C, and the resulting pellet containing plasma membranes was resuspended and diluted to a concentration of 5 mg/L. Native samples were boiled in Laemmli buffer at 70 °C for 10 min. For analysis of γ-ENaC cleavage fragments, samples were deglycosylated using PNGaseF according to the manufacturer’s instructions (NEB, Ipswich, USA) [24, 25]. First, samples were denaturated with a glycoprotein denaturing buffer. Samples were then incubated with glycobuffer, NP-40 and PNGaseF for 1 h at 37 °C. Subsequently, 20 µg of sample was loaded on an 8% (γ-ENaC) or 4–15% (α-, β-ENaC, NCC, NKCC2, and ROMK) polyacrylamide gel for electrophoresis. After transfer to nitrocellulose membranes (Amersham GE healthcare), the blocked blots were incubated with the primary antibodies outlined below. Signals were detected using fluorescent secondary antibody labelled with IRDye 800CW or IRDye 680RD and a fluorescence scanner (Licor Odyssey, Lincoln, USA). For loading control, total protein was measured using Revert Total Protein Stain (Licor, Lincoln, USA).

### Immunohistochemistry

For analysis of tissue expression of NKCC2, pNCC, γ-ENaC, and ROMK, kidneys were collected under control conditions or after 4 days on diuretic treatment. Paraffin-embedded formalin-fixed Sects. (1 µm) were deparaffinized with ethanol and rehydrated using standard protocols. Antigen retrieval was accomplished after heating for 5 min in antigen retrieval solution pH 6.1 (DAKO Deutschland GmbH, Hamburg, Germany) using a pressure cooker (Rommelsbacher, Germany). Kidney sections were blocked with avidin and biotin for each 15 min, followed by blocking for another 30 min with normal goat serum diluted 1:5 in 50 mM tris(hydroxymethyl)-aminomethane (Tris), pH 7.6, and 0.1 mL Tween 20%, supplemented with 5% (w/v) skim milk (Bio-Rad Laboratories, Munich, Germany). Sections were incubated overnight at 4 °C with the specific antibody (NKCC2 and NCC 1:500, γ-ENaC and ROMK 1:200, pNCC 1:2000) and subsequent washing in Tris buffer (50 mM Tris, pH 7.4, supplemented with 0.05% (v/v) Tween 20 (Sigma-Aldrich, Munich, Germany; 3 x). The secondary antibody (a biotinylated goat anti-rabbit, Vector Laboratories, Burlingame, CA, USA; 1:500) was applied for 30 min at room temperature. Sections were further processed using the VectaStain ABC kit according to the manufacturer’s instructions and DABImmPact (both Vector Laboratories) as substrate. Finally, the sections were counterstained in hematoxylin, dehydrated, and mounted for observation using a Zeiss upright microscope (Zeiss Axiovert 135 equipped with an Axiocam 208 color camera and software (ZEN 3.1) from Zeiss, Germany. Objectives used were LD Archoplan 20x/0,40 korr Ph2 and Plan-Apochromat 63x/ 1,40 Oil DIC from Zeiss, Germany). For each staining, four sections from at least two mice were analyzed at × 20 and × 63 magnification in order to be able to make a qualitative statement.

### Primary antibodies used in mouse samples

Antibodies against murine α- and β-ENaC were raised in rabbits against the amino acids 45–68 for α-ENaC and 617–638 for β-ENaC using a commercial service (Pineda, Berlin, Germany). Anti-γ-ENaC was purchased from Stressmarq (SPC-405, Viktoria, Canada). This antibody had been raised in rabbits against the C-terminal amino acids 634–655 of γ-ENaC. All antibodies were based on the peptide sequences first introduced and validated by Masilamani et al. [12] and used by many other researchers in the field such as in the work of Frindt et al. [25]. The antibodies against α- and β-ENaC had been affinity-purified while anti-γ-ENaC had been purified with protein A according to the manufacturer which was confirmed before [24]. Commercially available polyclonal antibodies were used to probe NKCC2 (SPC-401, Stressmarq), NCC (SPC-402, Stressmarq) and pNCC (p1311-53, Phosphosolutions). The antibody against ROMK was a kind gift from Johannes Loffing (University of Zürich, Switzerland).

### Quantitative PCR

Transcript levels of renin (*Ren*), mineralocorticoid receptor (*Nr3c2*), 11-β hydroxysteroid dehydrogenase 2 (*Hsd11b2*), sodium-hydrogen antiporter 3 (NH3) (*Slc9a3*), NKCC2 (*Slc12a1*), NCC (*Slc12a3*), α- (*Scnn1a*), β-(*Scnn1b*), and γ-ENaC (*Scnn1g*), ROMK (*Kcnj1*), SGK1 (*Sgk1*) and furin (*Furin*) were analyzed using quantitative real-time PCR with the LightCycler System (Roche Diagnostics, Mannheim, Germany). Kidney tissue was homogenized using the MagNa Lyser (Roche Diagnostics, Mannheim, Germany). Cleared cell lysate was transferred for further RNA purification (RNAeasy Mini Kit, Qiagen, Hilden, Germany). One microgram of total RNA was reverse-transcribed to cDNA (Transcriptor First Strand cDNA Synthesis Kit, Roche Diagnostics, Mannheim, Germany) with oligo(dT) primers according to the manufacturer’s protocol. Transcript levels of the target genes and the housekeeping genes GAPDH, ribosomal protein 13 (Rps13) and 18S-ribosomal RNA (Rn18s) were determined with the primer pairs as provided in Supplemental Table 1 [26–39]. PCR reactions were performed with 2 µL cDNA, 2.4 µL MgCl_2_ (4 mM), 1 µL primer mix (0.5 µM), 2 µL cDNA Master SYBR Green I mix (Roche Molecular Biochemicals, Mannheim, Germany), and DEPC-treated water, yielding a final volume of 20 µL. Melting point analysis and gel electrophoresis revealed a single product for all target and housekeeping genes.

Amplification was in the linear range as analyzed with serial dilutions of the amplicons. Crossing points of the products were determined from the maxima of the second derivative of the signal curve. The housekeeping gene Rn18s was selected for quantification, due to its performance throughout the diuretic treatment. Expression relative to the housekeeping gene Rn18s was calculated using the ΔC_t_ method [40, 41].

### Statistical analysis

Data are provided as means with SEM. Data were tested for normality with the Kolmogorov–Smirnov-Test, D’Agostino and Pearson omnibus normality test, and Shapiro–Wilk test. Variances were tested using the Bartlett’s test for equal variances. Accordingly, data were tested for significance with parametric or nonparametric ANOVA followed by Dunnett’s, Dunn’s, or Tukey’s multiple comparison post-test, paired or unpaired Student’s *t*-test, or Mann–Whitney *U*-test where applicable and Kaplan–Meier curves were tested with a Log-rank (Mantel-Cox) test using GraphPad Prism 10, GraphPad Software (San Diego, CA, www.graphpad.com). Densitometric analysis of the Western blots was done using Image Studio Version 3.1.4 and Empiria Studio Version 1.3.0.83 (Licor). A *p* value < 0.05 at two-tailed testing was considered statistically significant.

## Results

### Responses to acute diuretic administration

**Fig. 1:**
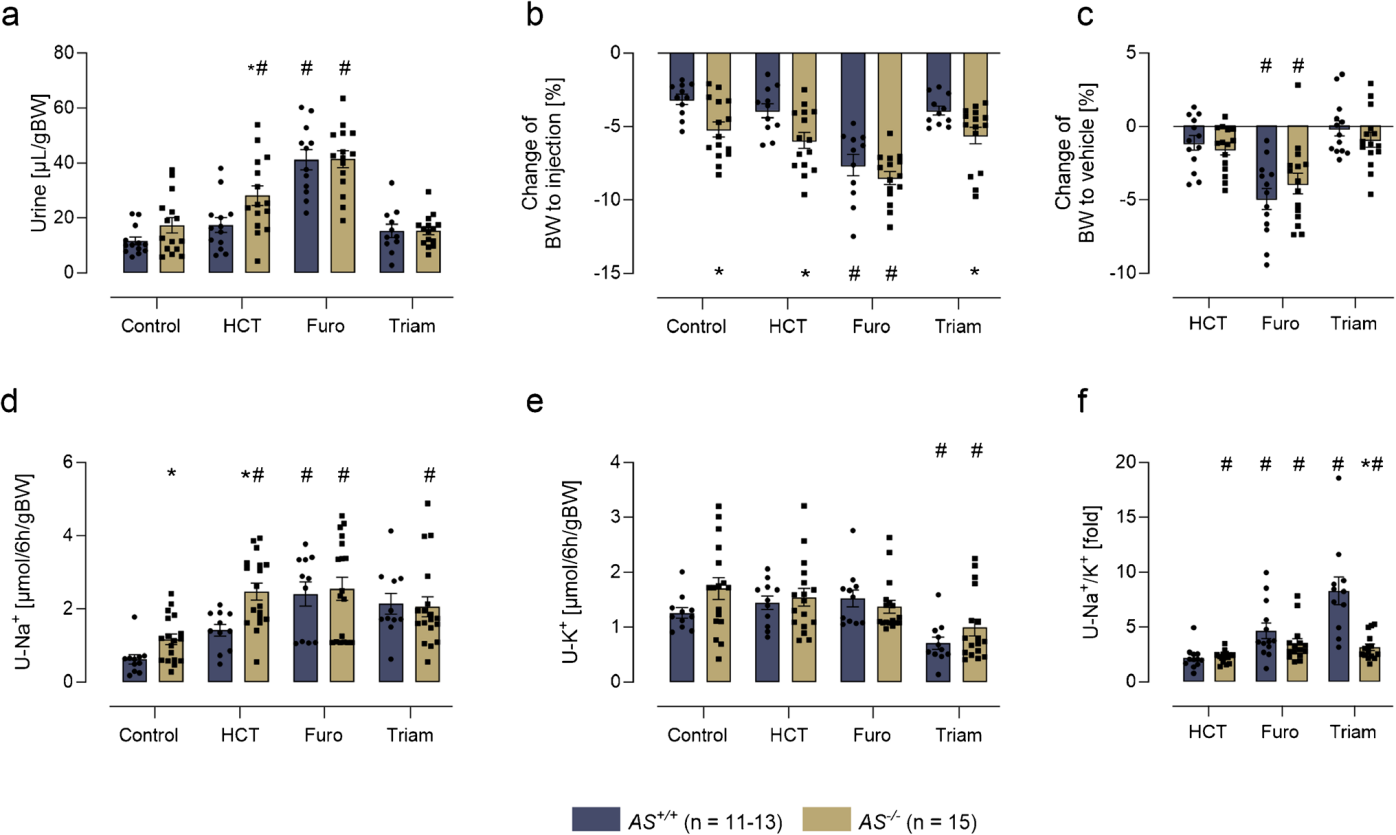
Responses to acute diuretic administration in *AS*^+*/*+^ and *AS*^*−/−*^ mice. **a** Urinary output after acute i.p. injection of vehicle, HCT, furosemide or triamterene under control conditions over 6 h. **b**, **c** Change of body weight compared to baseline and corrected for the change 6 h after vehicle injection. **d**, **e**, **f** Urinary sodium and potassium excretion as well as urinary sodium/potassium ratio over 6 h after injection. #*p* < 0.05 compared to vehicle treatment; *p < 0.05 between genotypes

*AS*^+*/*+^ and *AS*^*−/−*^ mice were administered with a single intraperitoneal bolus of vehicle, furosemide, HCT, or triamterene. In both genotypes, administration of furosemide resulted in the highest urine output and body weight loss (Fig. 1a–c). Furosemide and triamterene induced a comparable natriuresis in both genotypes, whereas natriuresis was stronger in *AS*^*−/−*^ mice after injection of HCT (Fig. 1d). As expected, urinary potassium excretion was reduced after administration of triamterene (Fig. 1e), however, the urinary sodium-to-potassium ratio as a measure of the ENaC-mediated sodium–potassium exchange in the principal cells was even stronger reduced in *AS*^*−/−*^ mice after triamterene treatment (Fig. 1f).

### Responses to chronic diuretic administration

**Fig. 2:**
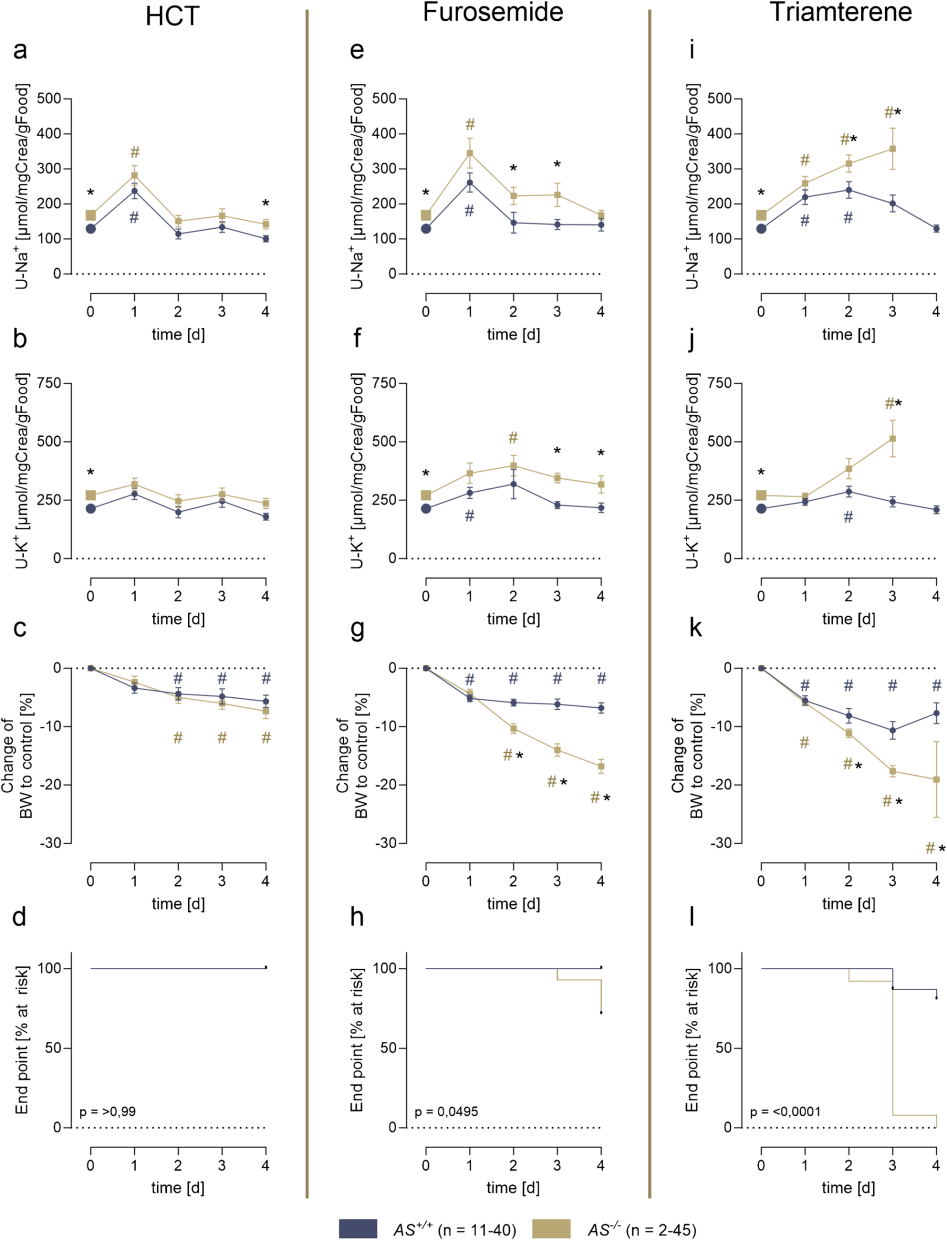
Responses to chronic diuretic administration in *AS*^+*/*+^ and *AS*^*−/−*^ mice. **a**, **e**, **i** Course of natriuresis during continuous diuretic treatment, normalized for creatinine and food intake per 24 h. **b**, **f**, **j** Course of potassium excretion during continuous diuretic treatment, normalized for creatinine and food intake per 24 h. **c**, **g**, **k** Change of body weight during the treatment, calculated as percentage change of baseline. **d**, **h**, **l** The Kaplan–Meier curves of the frequency of mice reaching the end point, defined by loss of more than 20% of body weight compared to baseline or deterioration, necessitating termination of the experiment. Note that the control values (**a**, **b**, **e**, **f**, **i**, **j**) at day 0 were pooled from all experimental series (indicated by larger symbols). #*p* < 0.05 compared to vehicle treatment; *p < 0.05 between genotypes

To assess the responses to chronic diuretic administration, a continuous treatment regimen was employed, comprising HCT (400 mg/L), furosemide (125 mg/L), and triamterene (200 mg/L) administered via the drinking water over 4 days without additional access to free water. The ingested doses of each diuretic were comparable between both gentoypes (Suppl. Fig. 1a, f, k). Under a control diet AS^*−/−*^ mice demonstrated a significantly increased natriuresis and kaliuresis (Fig. 2a, b) which was accompanied by a higher fluid and food intake and diuresis compared to *AS*^+*/*+^ mice (Suppl. Fig. 1b-d), as previously described [17].

Treatment with HCT demonstrated a comparable effect in both genotypes with a transient increased natriuresis at day 1 and adaptation during the following days (Fig. 2a). Urinary excretion of potassium stayed stable during treatment (Fig. 2b). The body weight declined in both genotypes modestly on day four (Fig. 2c, Suppl. Fig. 1e), and all mice completed the experiments (Fig. 2d).

Treatment with furosemide similarly increased natriuresis on day 1 in *AS*^+*/*+^ mice, which returned to the baseline values thereafter (Fig. 2e). Urinary potassium excretion also showed a temporary increase and reached the baseline values at the end of the treatment. (Fig. 2f). The effect on both natriuresis and kaliuresis was enhanced in *AS*^*−/−*^ mice during day 1 and the following days. In *AS*^+*/*+^ mice, food intake was stable, while fluid intake increased from day 1 on (Suppl. Fig. 1g, h). In contrast, food intake of *AS*^*−/−*^ mice decreased and the fluid intake did not increase. As anticipated, urine output was markedly elevated in both genotypes compared to the control values (Suppl. Fig. 1i). In *AS*^+*/*+^ mice body weight was reduced on day 1 and stabilized thereafter (Fig. 2g, Suppl. Fig. 1j). Conversely, *AS*^*−/−*^ mice continuously lost body weight, ultimately leading to the premature termination of the experiment in 29% of the mice (Fig. 2h).

To test the effect of prolonged inhibition of ENaC-mediated sodium transport, triamterene was administered. In *AS*^+*/*+^ mice, natriuresis increased on day 1 and returned to the baseline values thereafter (Fig. 2i). Urinary potassium excretion was minimally altered (Fig. 2j). Food and fluid intake remained stable while urine output increased slightly (Suppl. Fig. 2l-n). The effect on both urinary sodium and potassium excretion was enhanced in *AS*^*−/−*^ mice during day 1 and the following days. Notably, food and fluid intake and urine output exhibited a continuous decline in *AS*^*−/−*^ mice (Suppl. Fig. 1l-n). In *AS*^+*/*+^ mice body weight was reduced on day 1 and stabilized in most of the mice thereafter (Fig. 2k, Suppl. Fig. 1o). However, 17% of the *AS*^+*/*+^ mice reached the endpoint for termination of the experiment at day 4. Conversely, all *AS*^*−/−*^ mice continuously lost body weight and deteriorated, ultimately leading to the premature termination of the experiment in all of the mice (100%) (Fig. 2h).

### Changes of pH, hematocrit, plasma electrolytes, urea, and aldosterone concentrations

**Fig. 3:**
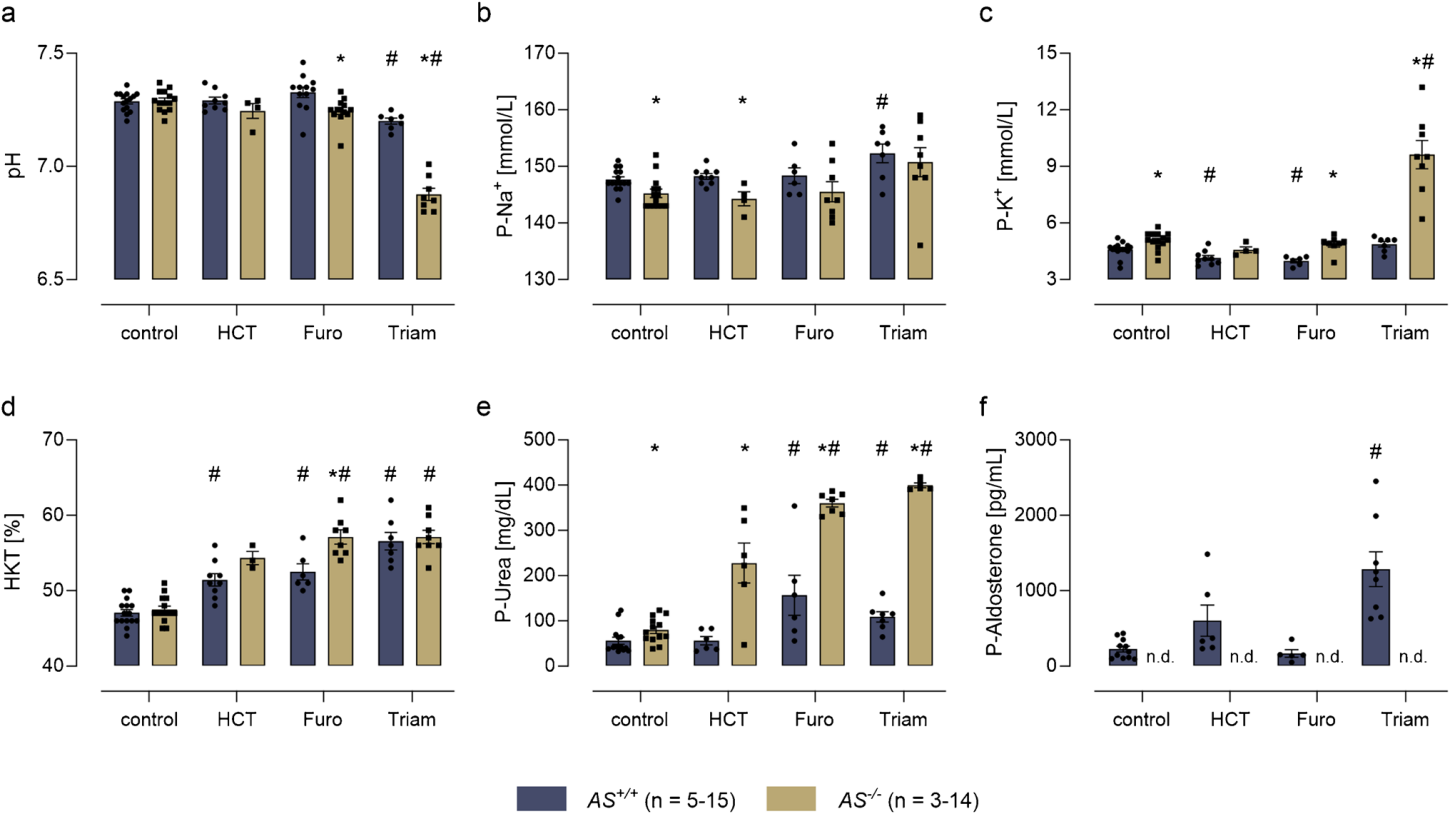
Changes of pH, hematocrit, plasma electrolytes, urea and aldosterone concentrations in *AS*^+*/*+^ and *AS*^*−/−*^ mice. Venous blood pH (**a**), plasma sodium (**b**) and potassium concentrations (**c**), hematocrit (**d**), plasma urea (**e**), and aldosterone concentrations (**f**) under control conditions and on the last day of diuretic administration. Please note that there was no detectable (n.d.) plasma aldosterone in *AS*^*−/−*^ mice. #*p* < 0.05 compared to vehicle treatment; *p < 0.05 between genotypes

Under control conditions, pH was comparable between the genotypes and remained stable in *AS*^+/+^ mice under chronic diuretic treatment. In *AS*^*−/−*^ mice, pH was stable during treatment with HCT and furosemide, however, under triamterene *AS*^*−/−*^ mice developed severe metabolic acidosis with a pH < 7.0 and a bicarbonate concentration < 13 mM (Fig. 3a). Plasma sodium concentrations slightly decreased in *AS*^*−/−*^ mice under control conditions and HCT treatment, a difference that disappeared under furosemide and triamterene treatment (Fig. 3b). In *AS*^+*/*+^ mice, plasma potassium concentrations decreased under HCT and furosemide treatment and remained stable under triamterene treatment (Fig. 3c). Plasma potassium concentrations were slightly increased in *AS*^*−/−*^ mice under control conditions and *AS*^*−/−*^ mice developed massive hyperkalemia > 9 mM under triamterene treatment. Under control conditions, hematocrit was similar in both genotypes and significantly increased under furosemide and triamterene treatment in both genotypes (Fig. 3d). In *AS*^+*/*+^ mice, plasma urea concentrations increased under furosemide and triamterene treatment and remained stable under HCT treatment (Fig. 3e). Plasma urea concentrations were slightly increased in *AS*^*−/−*^ mice under control conditions and massively increased during any diuretic treatment with highest values observed under triamterene treatment, suggesting development of acute kidney injury. Plasma aldosterone concentrations indicated a tendency towards increased values under HCT treatment and a significant increase by ~ sixfold under triamterene treatment while in *AS*^*−/−*^ mice aldosterone was not detectable (Fig. 3f).

### Expression pattern of γ-ENaC, NKCC2, NCC, and ROMK in kidney tissue under chronic diuretic administration

**Fig. 4:**
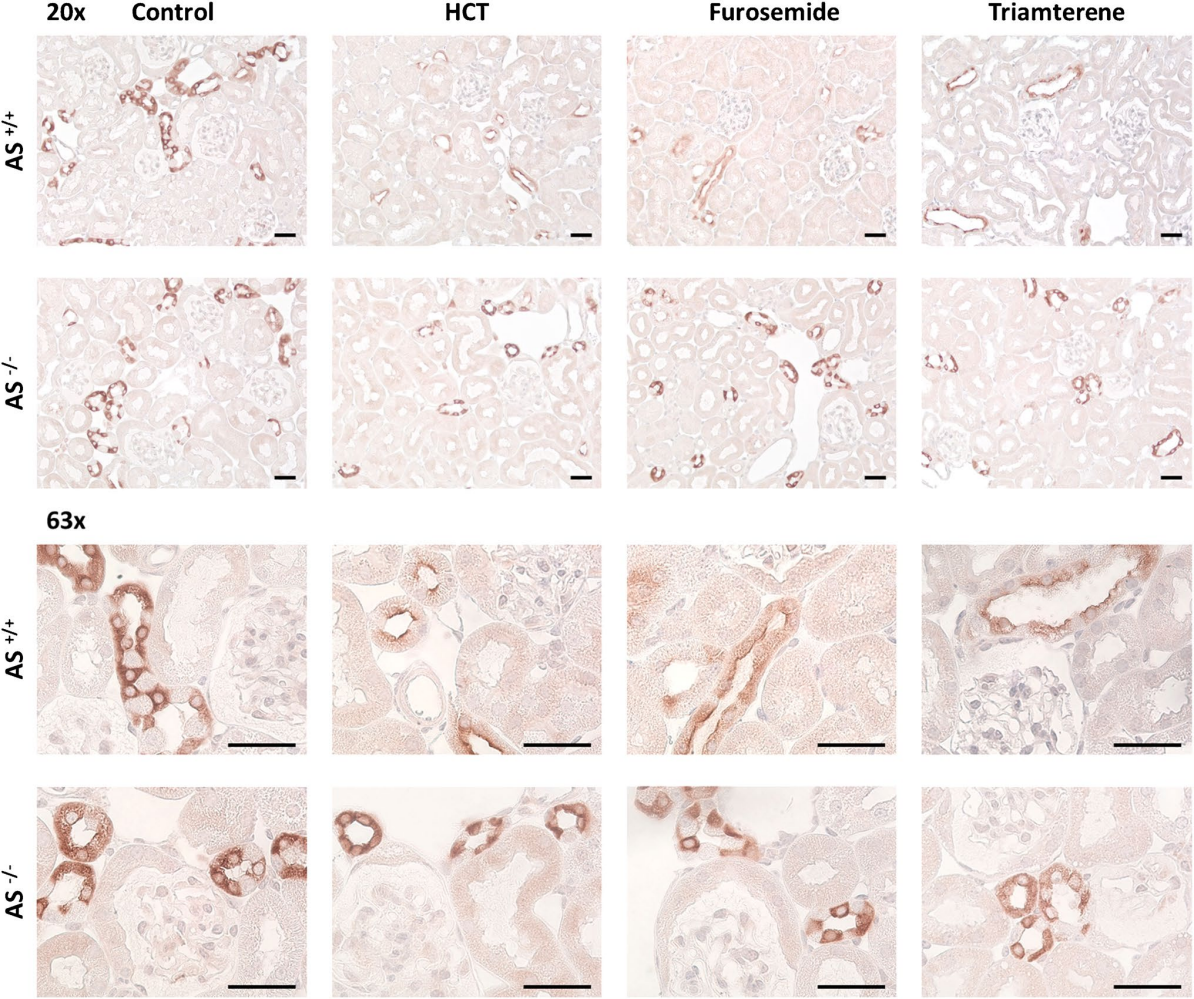
Expression pattern of γ-ENaC in kidney tissue under control conditions and chronic diuretic administration in *AS*^+*/*+^ and *AS*^*−/−*^ mice. Representative staining of kidney sections stained for γ-ENaC at 20- (upper panels) and 63-fold (lower panels) magnification (scale 20 µm)

**Fig. 5:**
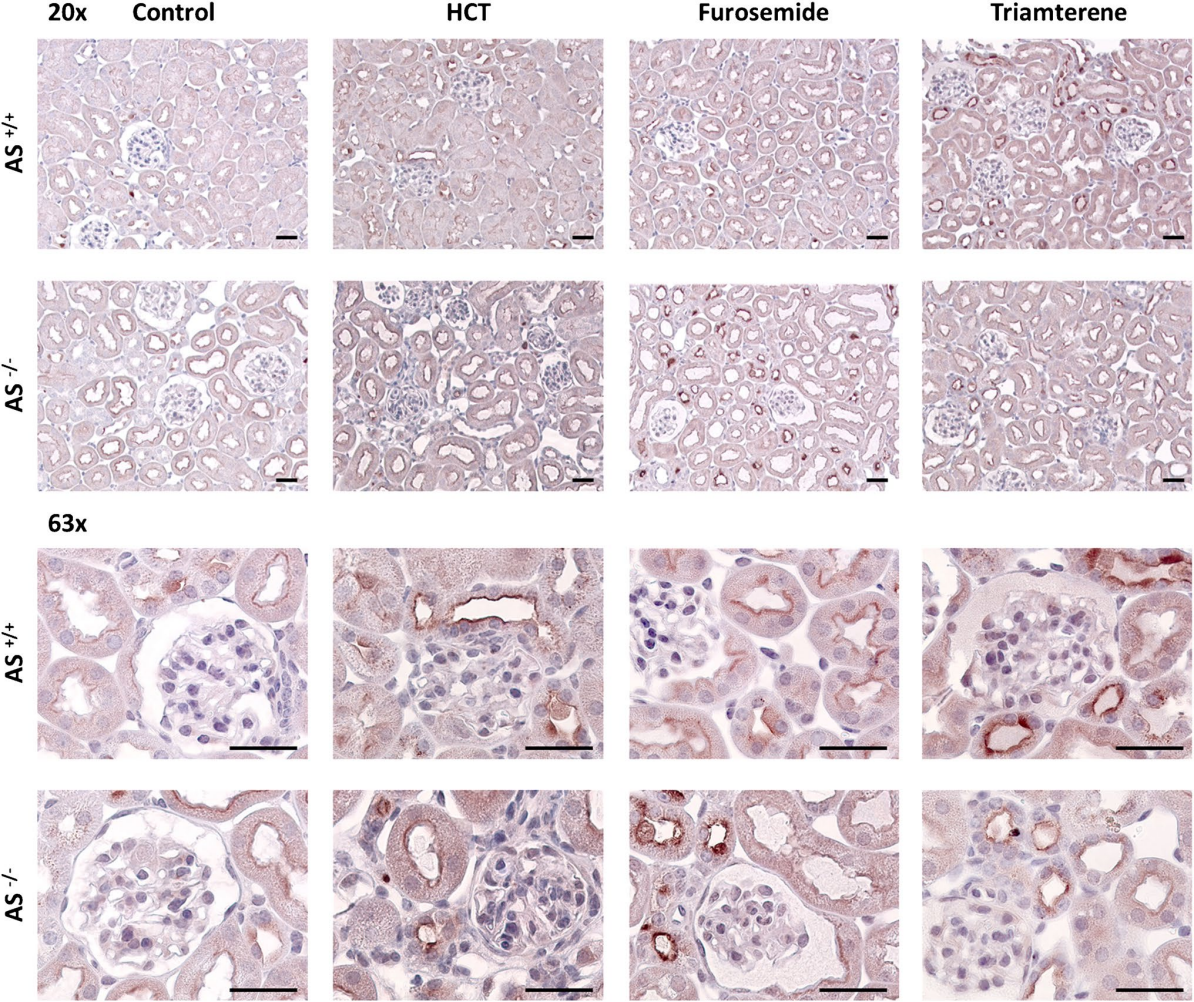
Expression pattern of ROMK in kidney tissue under control conditions and chronic diuretic administration in AS^+/+^ and AS^*−*/*−*^ mice. Representative staining of kidney sections stained for ROMK 20- (upper panels) and 63-fold (lower panels) magnification (scale 20 µm)

Under control conditions, immunohistochemical γ-ENaC staining followed a predominantly intracellular pattern in both genotypes (Fig. 4). Under chronic administration of all diuretics, there was an increased abundance of γ-ENaC at the apical plasma membrane of *AS*^+*/*+^ mice, known as apical targeting in the setting of hyperaldosteronism [42, 43]. In *AS*^*−/−*^ mice, however, apical targeting was strongly reduced (Fig. 4), suggesting a defect in ENaC trafficking. The immunohistochemical staining for NKCC2, NCC and phosphorylated NCC (pNCC) showed an apical pattern already under control conditions in both genotypes and under chronic diuretic administration this pattern did not change appreciably (Suppl. Fig. 2—4). In addition to the investigation of the expression of the various sodium transporters inhibited by the diuretics used, expression of the potassium channel ROMK was analyzed. As shown in Fig. 5, ROMK expression was apical and seemed to be similar in both genotypes under control conditions. Under chronic diuretic administration, ROMK expression tended to be increased at the apical membrane in both genotypes.

### Expression of ENaC subunits and proteolytic processing in kidney lysates under chronic diuretic treatment

**Fig. 6:**
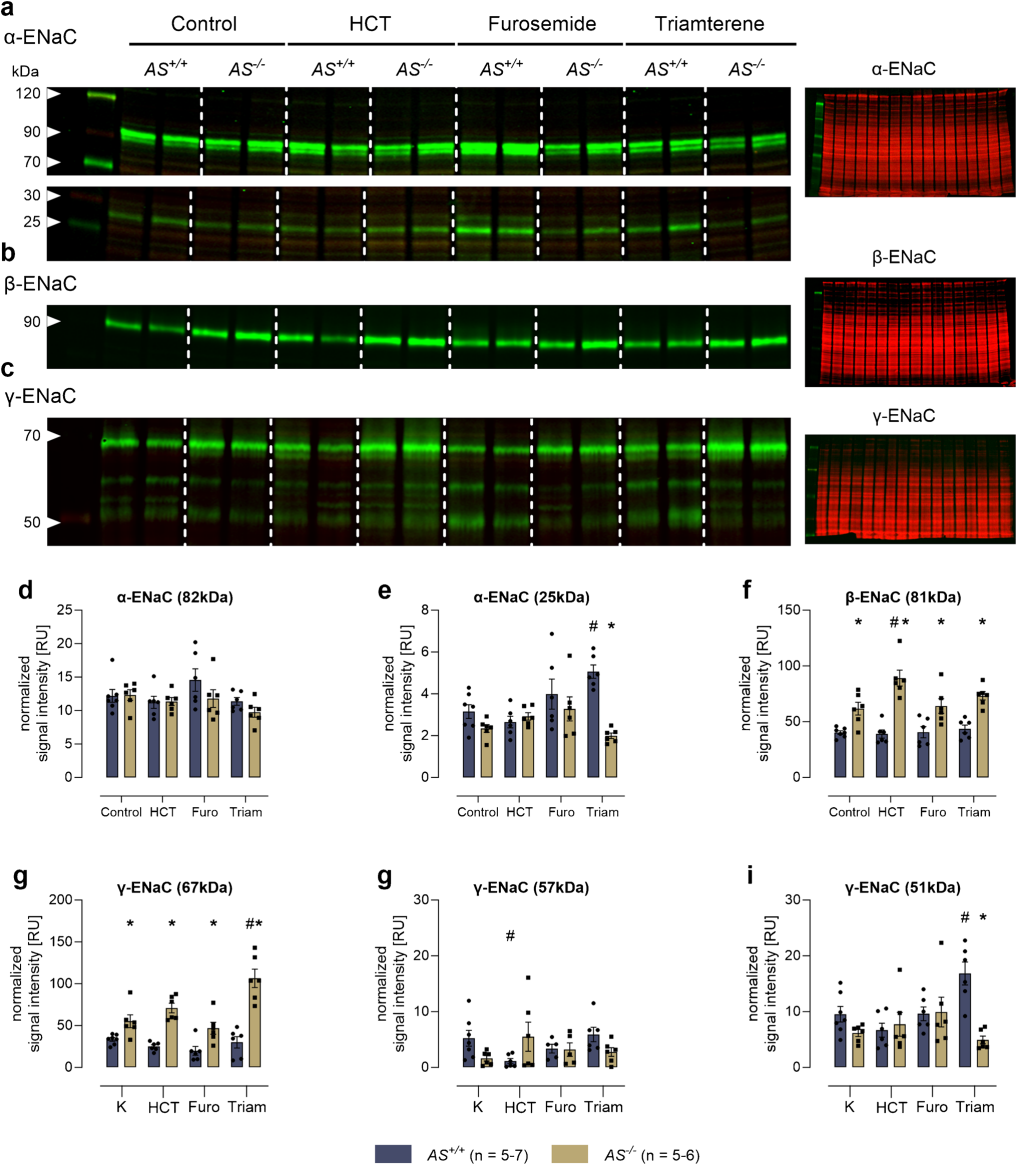
Expression of ENaC subunits and proteolytic processing in kidney lysates under control conditions and chronic diuretic treatment in *AS*^+*/*+^ and *AS*^*−/−*^ mice. Representative Western blot showing the expression of α-ENaC (**a**), β -ENaC (**b**), and γ-ENaC (**c**) in a plasma membrane preparation of kidney cortex lysates before (control) and after diuretic treatment. Total protein stain as a loading control on the right side each. **d**–**i** Densitometry of the obtained bands normalized for total protein content of each lane. #*p* < 0.05 compared to vehicle treatment; *p < 0.05 between genotypes

In Western blot analyses of kidney cortex lysates, two bands for α-ENaC at 82 and 25 kDa corresponding to full-length and a cleavage product after distal cleavage were identified (Fig. 6a, d, e). There was only a single band for β-ENaC at 81 kDa corresponding to the full-length subunit (Fig. 6b, f). For γ-ENaC there were three bands in deglycosylated samples at 67, 57, and 51 kDa, corresponding to full-length, proximally and distally cleaved fragments (Fig. 6c, g–i). Under control conditions, there was no difference in the expression of α-ENaC and its cleavage product between both genotypes while *AS*^*−/−*^ mice showed a higher expression of β-ENaC and the uncleaved γ-ENaC at 67 kDa (Fig. 6a–g). This pattern remained unaltered under treatment with furosemide and HCT. However, under triamterene treatment there was a significant increase in the expression of the cleavage products of α- and γ-ENaC in *AS* ^+*/*+^ mice which was not seen in *AS*^*−/−*^ mice (Fig. 6e, i). Conversely, in *AS*^*−/−*^ mice there was only an increase in the expression of uncleaved γ -ENaC at 67 kDa (Fig. 6g).

### Expression of NKCC2, NCC and ROMK in kidney lysates under chronic diuretic treatment

Under control conditions, expression of NKCC2 and NCC were similar in both genotypes (Suppl. Fig. 5) and under diuretic treatment, the expression of both transporters tended to increase in *AS*^+*/*+^ mice. In *AS*^*−/−*^ mice, the expression of NKCC2 was particularly reduced in *AS*^*−/−*^ mice under triamterene treatment (Suppl. Fig. 5a, d) and expression of NCC was significantly reduced under any diuretic treatment (Suppl. Fig. 5b, e). With regard to expression of ROMK, there was no difference in both genotypes under control conditions, but a significantly lower expression in *AS*^*−/−*^ mice under HCT and triamterene treatment (Suppl. Fig. 6).

### Effect of chronic diuretic administration on the mRNA expression of the sodium transporters and their regulators

*AS*^*−/−*^ mice showed a higher mRNA expression of renin under control treatment and triamterene treatment compared with *AS*^+*/*+^ mice (Suppl. Fig. 7a). The mRNA expression of NKCC2, NCC, the ENaC subunits and sodium-hydrogen exchanger NHE3 as well ROMK were similar in both genotypes under control conditions. This was also true for the mRNA expression of the mineralocorticoid receptor, furin, serum-and-glucocorticoid-dependent kinase 1 (SGK1), and 11-β hydroxysteroid dehydrogenase 2 (Suppl. Fig. 7b-l). Under chronic diuretic administration, the expression of these transporters and regulatory factors showed great variation, and there was no clear pattern in *AS*^+*/*+^ mice. In *AS*^*−/−*^ mice, mRNA expression of NKCC2, NCC, ENaC subunits, and ROMK tended to decrease under chronic diuretic administration (Suppl. Fig. 7b-l).

## Discussion

The present study demonstrates that aldosterone plays a pivotal role in the development of diuretic resistance and tolerance in wild-type mice. In contrast, mice lacking aldosterone synthase were intolerant to repeated doses of furosemide and particularly of triamterene, leading to severe weight loss, hyperkalemia and acidosis. These findings indicate the significance of aldosterone-regulated epithelial sodium channel (ENaC) activity and potassium balance in the adaptation to diuretic treatment, particularly during ENaC blockade with triamterene. In wild-type mice, diuretic resistance to triamterene was paralleled by increased apical abundance of γ-ENaC and by stimulation of proteolytic processing of both the α- and γ-subunit. Both effects act in concert to counteract ENaC inhibition by increasing channel abundance (N) and open probability (Po) and are known to be stimulated by aldosterone via effects on trafficking and stimulation of proteolytic processing by serine proteases. One of the serine proteases involved in proteolytic ENaC processing during triamterene treatment has been identified to be prostasin which is expressed as membrane-anchored serine protease in the tubulus [44]. Mice expressing zymogen-locked but not enzymatically active prostasin were similarly intolerant to triamterene treatment and developed the same phenotype as *AS*^*−/−*^ mice [22]. Kidney lysates expression of cleaved γ-ENaC was reduced in mice expressing zymogen-locked prostasin compared to wild-type mice [22, 45]. *AS*^*−/−*^ mice also recapitulated the phenotype of mice deficient for the intracellular kinases SGK1 and mTORC2 which were also intolerant to ENaC inhibition by triamterene [20, 21]. Both kinases are involved in a cascade regulating ENaC trafficking and membrane abundance by phosphorylating the ubiquitin ligase Nedd4-2 that targets ENaC subunits for proteasomal degradation.

One mechanism of diuretic resistance is the upregulation of the respective sodium transporter at the site of inhibition. G.H. Kim found that the protein expression of each NKCC2, NCC, and ENaC was increased under respective treatment with furosemide, HCT and amiloride in kidney lysates from Sprague–Dawley rats [5]. In our study we could not reproduce the increased protein expression of the sodium transporters by Western blot. Using immunohistochemistry, we found that the apical membrane abundance of ENaC was clearly enhanced in *AS*^+*/*+^ mice (Fig. 4) while we did not see a clear difference for NKCC2 and NCC, both of which had a high membrane abundance under control conditions (Suppl. Figs. 2 and 3). The upregulation of sodium transporters at the site of inhibition might be mediated by a drop in intracellular solute concentration. For ENaC intracellular sodium concentration has been found to be a potent regulator of ENaC by stimulating proteolytic processing [46]. For both NKCC2 and NCC, intracellular chloride has been reported to be a potent regulator of channel phosphorylation and membrane abundance via the action of the chloride-sensitive with-no-lysine kinases 1 and 4 (WNK1/4) [47, 48]. Inhibition of NKCC2 and NCC would therefore reduce intracellular chloride and stimulate channel phosphorylation via WNK1/4, thereby increasing their membrane abundance. Using a phospho-specific antibody, we could not find a clear increase in pNCC expression using immunohistochemistry (Suppl. Fig. 4); however, a subtle difference cannot be ruled out. Noteworthy, *AS*^*−/−*^ mice were completely tolerant to HCT treatment, indicating that NCC upregulation was not impaired and the contribution of ENaC was not decisive. This fitted to the finding that aldosterone was not appreciably stimulated in HCT-treated *AS*^+*/*+^ mice which corresponded to the absence of hyperkalemia (Fig. 3f). Overall, IHC analyses were limited by the lack of data on the spatial resolution of the sodium transporters in various tubule segments such as ENaC and ROMK, which would have required confocal microscopy and co-staining of tubulus segment specific markers.

The results confirm that *AS*^*−/−*^ mice have salt-wasting which is compensated under control conditions due to increased food and fluid intake, activation of the renin-angiotensin-system [49] and increased cortisol secretion [16]. Elegant electrophysiological studies with microdissected tubuli indicated that *AS*^*−/−*^ mice have normal ENaC activity at the late distal convoluted tubule/connecting tubule (DCT2/CNT) while ENaC activity was reduced distally in the collecting duct (CCD) [50]. A low sodium diet had little effect on ENaC-mediated sodium transport in the DCT2/CNT but failed to upregulate ENaC transport in the CNT/CCD, indicating aldosterone-dependency of ENaC activity in these distal parts of the nephron. Our results indicate that ENaC activity in CNT/CCD becomes relevant during pharmacological ENaC inhibition and is required for the development of diuretic resistance to furosemide and triamterene in *AS*^+*/*+^ mice. Our studies on ENaC expression in *AS*^*−/−*^ mice revealed profound differences to *AS*^+*/*+^ mice. First, at the protein level, *AS*^*−*/*−*^ mice had increased abundance of β- and uncleaved γ-ENaC but reduced expression of furin-cleaved α- and fully cleaved γ-ENaC under triamterene treatment (Fig. 6). Second, *AS*^*−/−*^ mice had reduced apical targeting of ENaC during diuretic treatment (Fig. 4). These findings indicate that aldosterone is required for increasing ENaC membrane abundance and proteolytic processing in the CNT/CCD. Hence, ENaC in CNT/CCD serves as a compensatory pool which becomes relevant under conditions such as low sodium diet or treatment with furosemide or triamterene. The data suggest that *AS*^*−/−*^ mice suffer from a deficit of ENaC maturation and trafficking which has been similarly reported by Todkar et al. [49]. It is tempting to speculate that reduced membrane abundance leads to blunted extracellular proteolytic processing; however, γ-ENaC can also be fully cleaved by intracellular serine proteases such as TMPRSS2 (transmembrane protease serine subtype 2) [51, 52]. The full spectrum of ENaC dysregulation in *AS*^*−/−*^ mice is expected to involve different mechanisms and remains to be investigated.

In *AS*^*−/−*^ mice, triamterene treatment caused massive hyperkalemia exceeding values > 9 mM and severe metabolic acidosis. A similar degree of hyperkalemia and acidosis was shown to develop in mice with inducible deletion of γ-ENaC within 2 days [53]. These findings underscore the essential role of ENaC-mediated sodium uptake for potassium and hydrogen ion secretion in the distal nephron. In contrast, *AS*^*−/−*^mice did not develop hyperkalemia and acidosis during treatment with HCT or furosemide because the driving forces were not altered. As already shown by Todkar et al. [49], there was no discernible difference in the expression of the ROMK between *AS*^+*/*+^ mice and *AS*^*−/−*^ mice under control conditions. This fits to the current notion that ROMK expression is not regulated by aldosterone [54]. Surprisingly, despite massive hyperkalemia, ROMK protein expression was rather reduced in HCT- and triamterene-treated *AS*^*−/−*^ mice (Suppl. Fig. 6) which is expected to aggravate potassium retention and hyperkalemia. Currently, we do not have an explanation for this. Finally, acidosis could also contribute to reduced potassium secretion as ROMK activity is highly pH-sensitive [55]. In agreement with current knowledge, NCC was downregulated on a protein level in *AS*^*−/−*^ mice during triamterene treatment (Suppl. Fig. 5), reflecting the inhibitory effect of hyperkalemia on NCC expression under conditions of reduced or absent ENaC function [49, 53]. The inhibitory effect of hyperkalemia is mediated by WNK1/4 and aims to shift sodium downstream for stimulation of ENaC-mediated potassium secretion [56]. However, downregulation of NCC is expected to exacerbate sodium loss and promote the observed phenotype in *AS*^*−/−*^ mice lacking upregulation of ENaC activity under triamterene treatment.

Diuretic resistance is a common finding in clinical practice and can negatively impact the management of patients with sodium and water retention such as those with congestive heart failure [57]. Our results indicate that ENaC blockade might aid in overcoming diuretic resistance. This could be done by adding ENaC blockers such as amiloride or triamterene to a diuretic regimen with furosemide. Our data also suggest that adding a MR blocker such as spironolactone or eplerenone could also be beneficial [58]. In addition, the potassium-sparing effect of ENaC blockade might oppose the potassium-wasting effect of furosemide or HCT. Of note, spironolactone is considered standard treatment in patients with heart failure and shown to improve heart failure outcomes and most importantly to reduce mortality as demonstrated in the RALES study [59]. A rapid initiation of low dose spironolactone could therefore be beneficial to prevent the development of diuretic resistance. However, in the patient with chronic kidney disease and reduced GFR < 30 mL/min/1.73m^2^ ENaC blockade is problematic and bears the risk of provoking life-threatening hyperkalemia [60], limiting this approach in this population.

In conclusion, we show that aldosterone plays an essential role in the development of diuretic resistance to blockade of NKCC2 and ENaC, but not to NCC. In the light of the recent developments of aldosterone synthase inhibitors and MR antagonists [61, 62], new therapeutic options are emerging to overcome diuretic resistance caused by elevated aldosterone.

## Supplementary Information

Below is the link to the electronic supplementary material.

Supplementary file1 (DOCX 3.90 MB)

## Data Availability

Data will be shared upon reasonable request.
