# Supplementary material for: Impact of aldosterone deficiency on the development of diuretic resistance in mice
Source: Pflugers Arch. 2025 Apr 12;477(6):827–40. doi: 10.1007/s00424-025-03082-8 (PMC12092488; doi:10.1007/s00424-025-03082-8)
Supplement: Supplementary file 1 — Supplementary file1 (DOCX 3.90 MB) [file 424_2025_3082_MOESM1_ESM.docx]

**Supplement:**

**Supplemental Figure 1 Food and fluid intake as well as urine output and change of body weight control conditions and diuretic treatment in *AS^+/+^* and *AS^-/-^* mice**


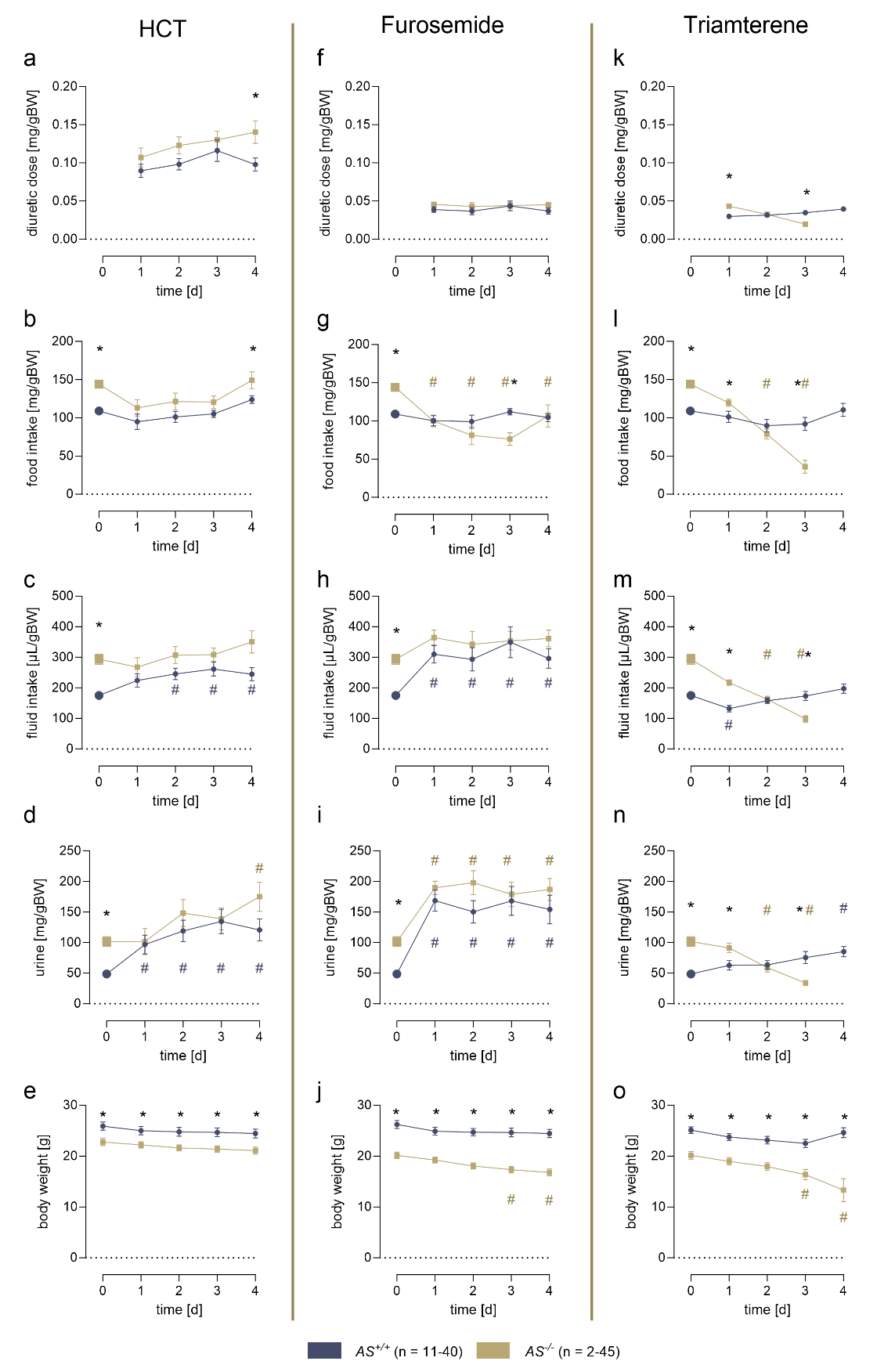


Diuretic dose (a, f, k), food intake (b, g, l), fluid intake (c, h, m), urine output (d, i, n) over 24h during control conditions and diuretic treatment over 4 days. Course of body weight during diuretic treatment over 4 days (e, j, o).

Note that the control values at day 0 (except for body weight) were pooled from all experimental series (indicated by larger symbols).

# p<0.05 compared to vehicle treatment, * p<0.05 between genotypes

**Supplemental Figure 2: Expression pattern of NKCC2 in kidney tissue under control conditions and chronic diuretic administration in *AS^+/+^* and *AS^-/-^* mice**


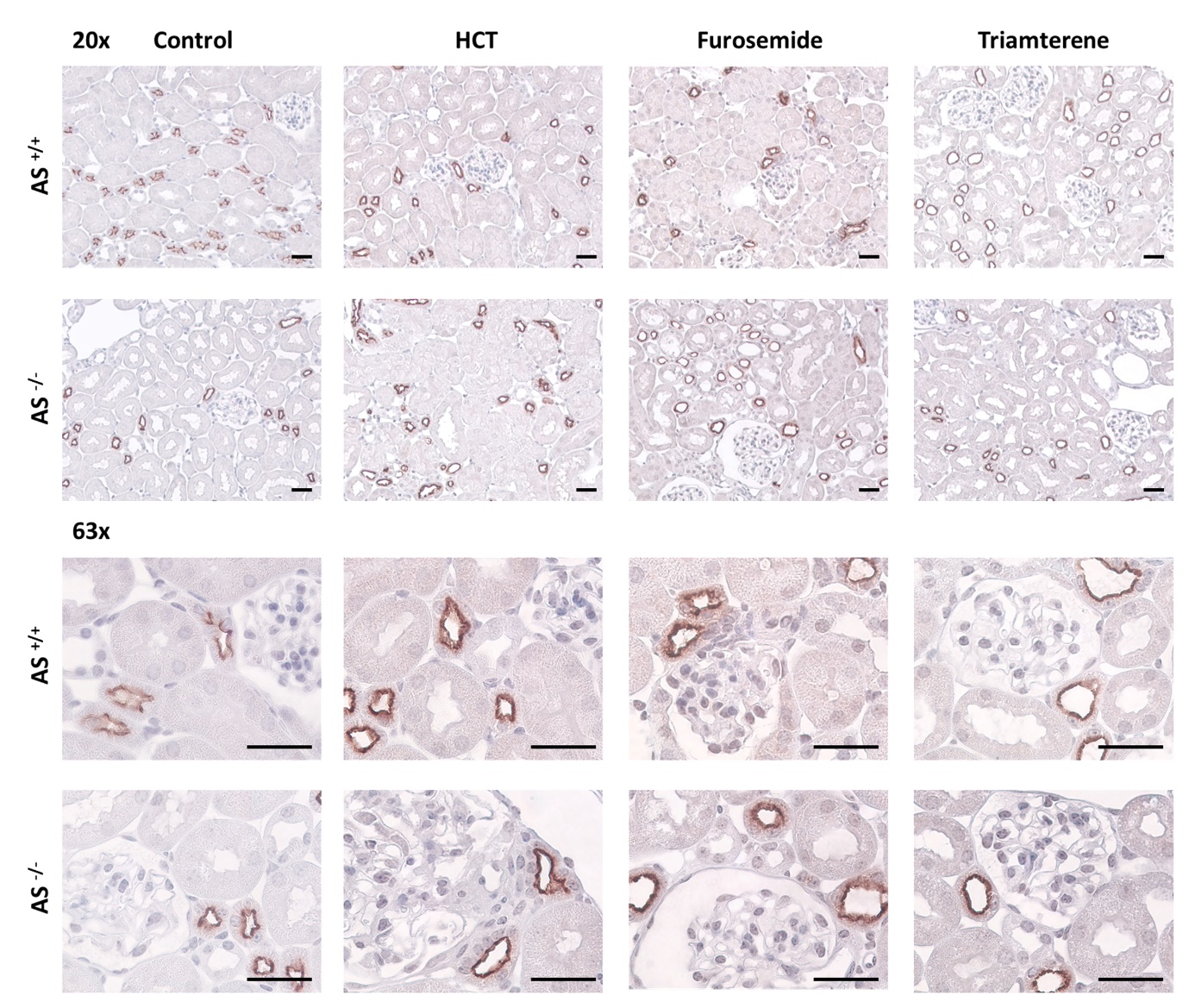


Representative staining of kidney sections stained for NKCC2 at 20- (upper panels) and 63-fold (lower panels) magnification. (scale 20µm)

**Supplemental Figure 3: Expression pattern of NCC in kidney tissue under control conditions and chronic diuretic administration in *AS^+/+^* and *AS^-/-^* mice**


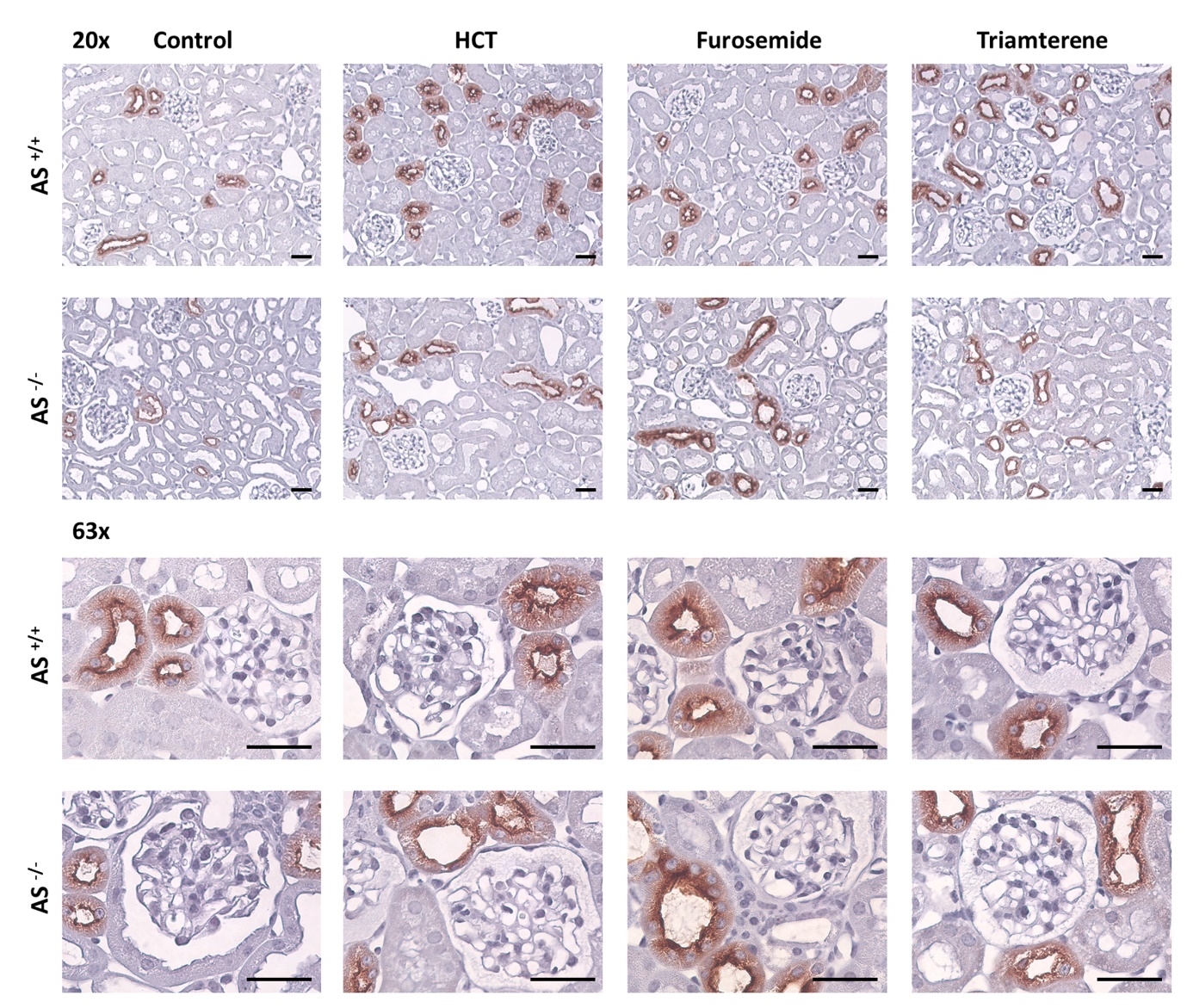


Representative staining of kidney sections stained for NCC at 20- (upper panels) and 63-fold (lower panels) magnification. (scale 20µm)

**Supplemental Figure 4: Expression pattern of phosphorylated NCC in kidney tissue under control conditions and chronic diuretic administration in *AS^+/+^* and *AS^-/-^* mice**


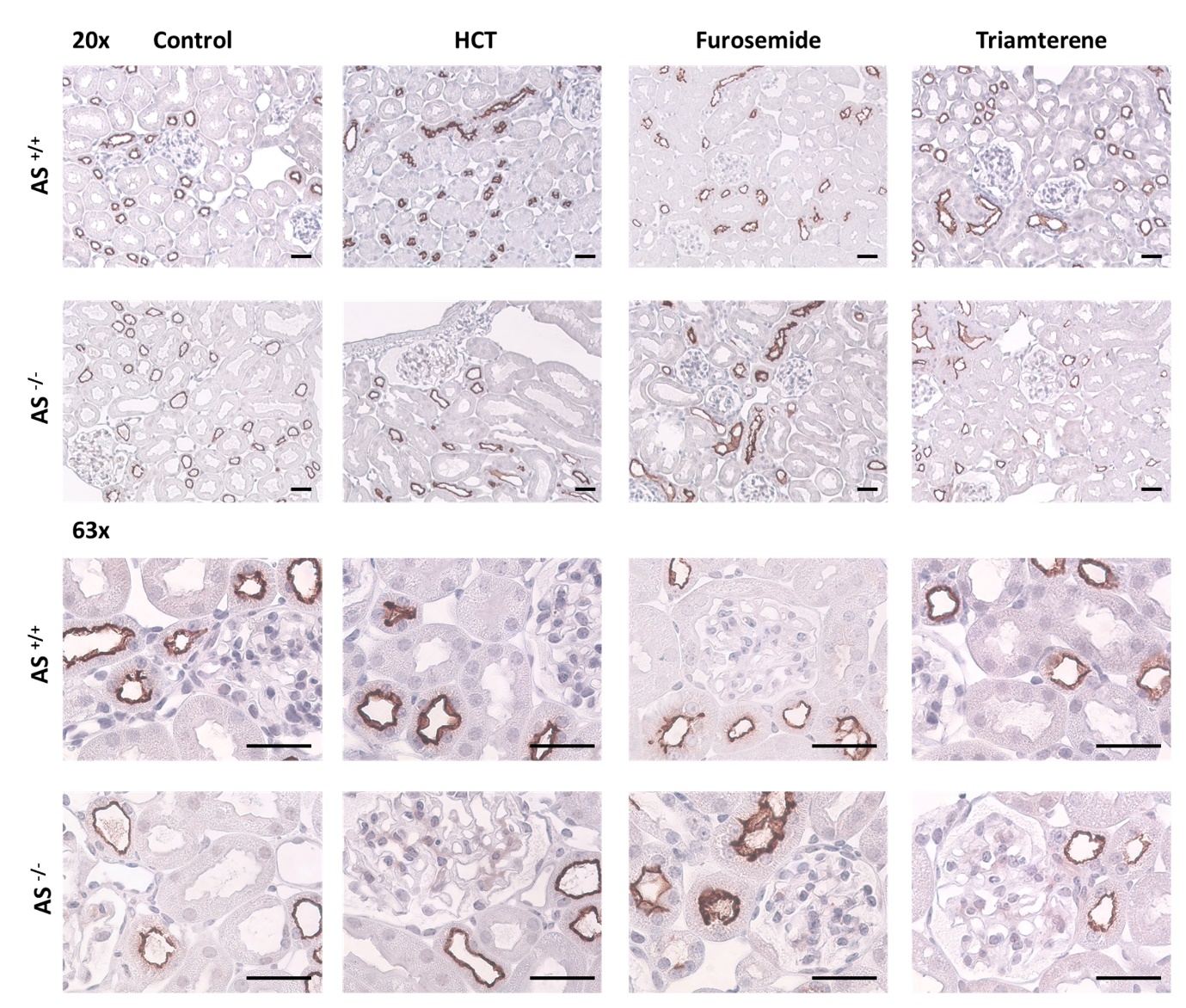


Representative staining of kidney sections stained for phosphorylated NCC at 20- (upper panels) and 63-fold (lower panels) magnification. (scale 20µm)

**Supplemental Figure 5: Expression of NKCC2 and NCC in kidney lysates under control conditions and diuretic treatment in *AS^+/+^* and *AS^-/-^* mice**


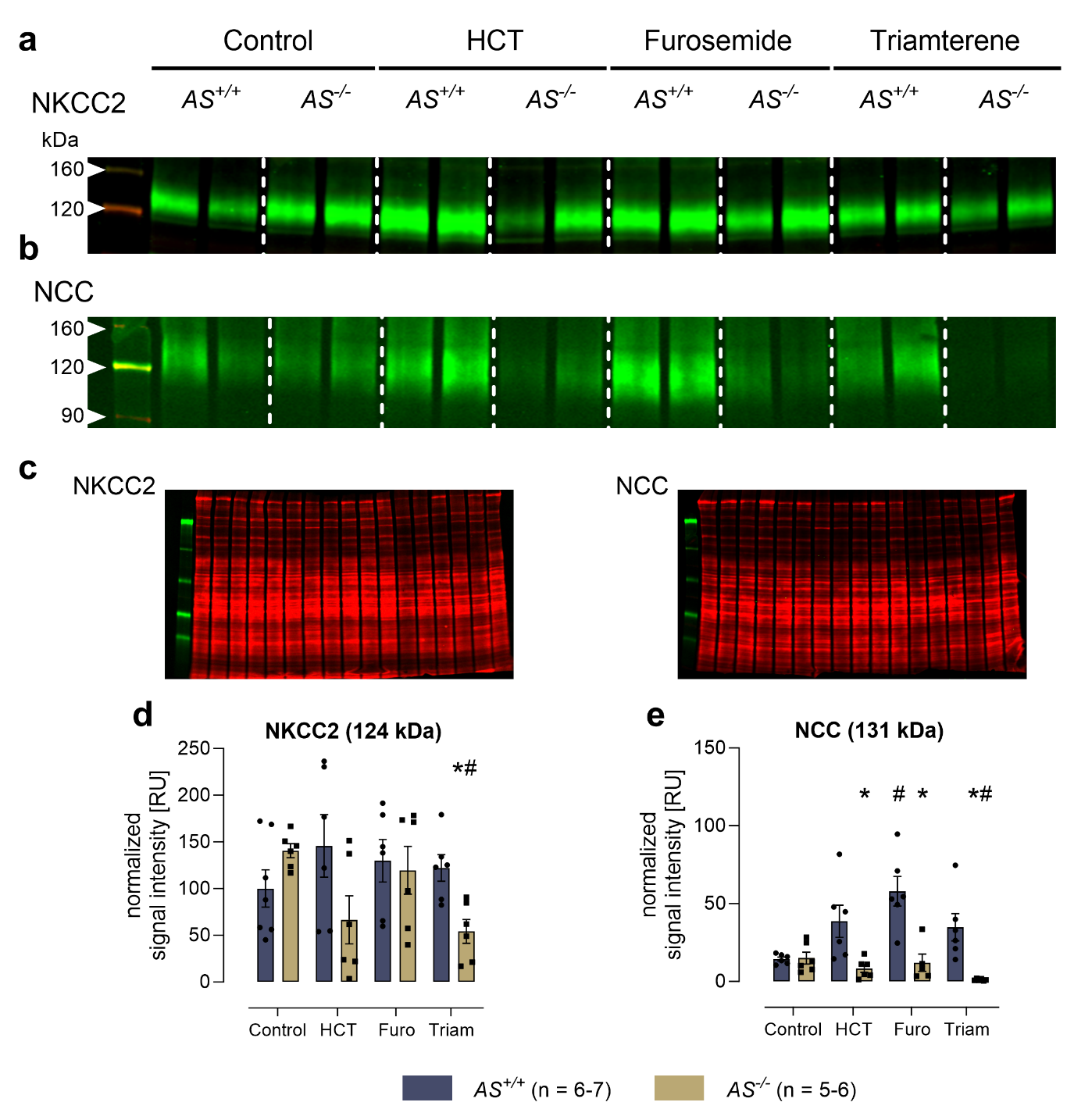


a Representative Western blot showing the expression of NKCC2 in a plasma membrane preparation of kidney cortex lysates before (control) and after diuretic treatment

b Representative Western blot showing the expression of NCC in a plasma membrane preparation of kidney cortex lysates before (control) and after diuretic treatment

c Total protein stain as a loading control

d, e Densitometry of the obtained bands normalized for total protein content of each lane

# p<0.05 compared to vehicle treatment, * p<0.05 between genotypes

**Supplemental Figure 6: Protein expression of ROMK in kidney lysates under control conditions and diuretic treatment in *AS^+/+^* and *AS^-/-^* mice**


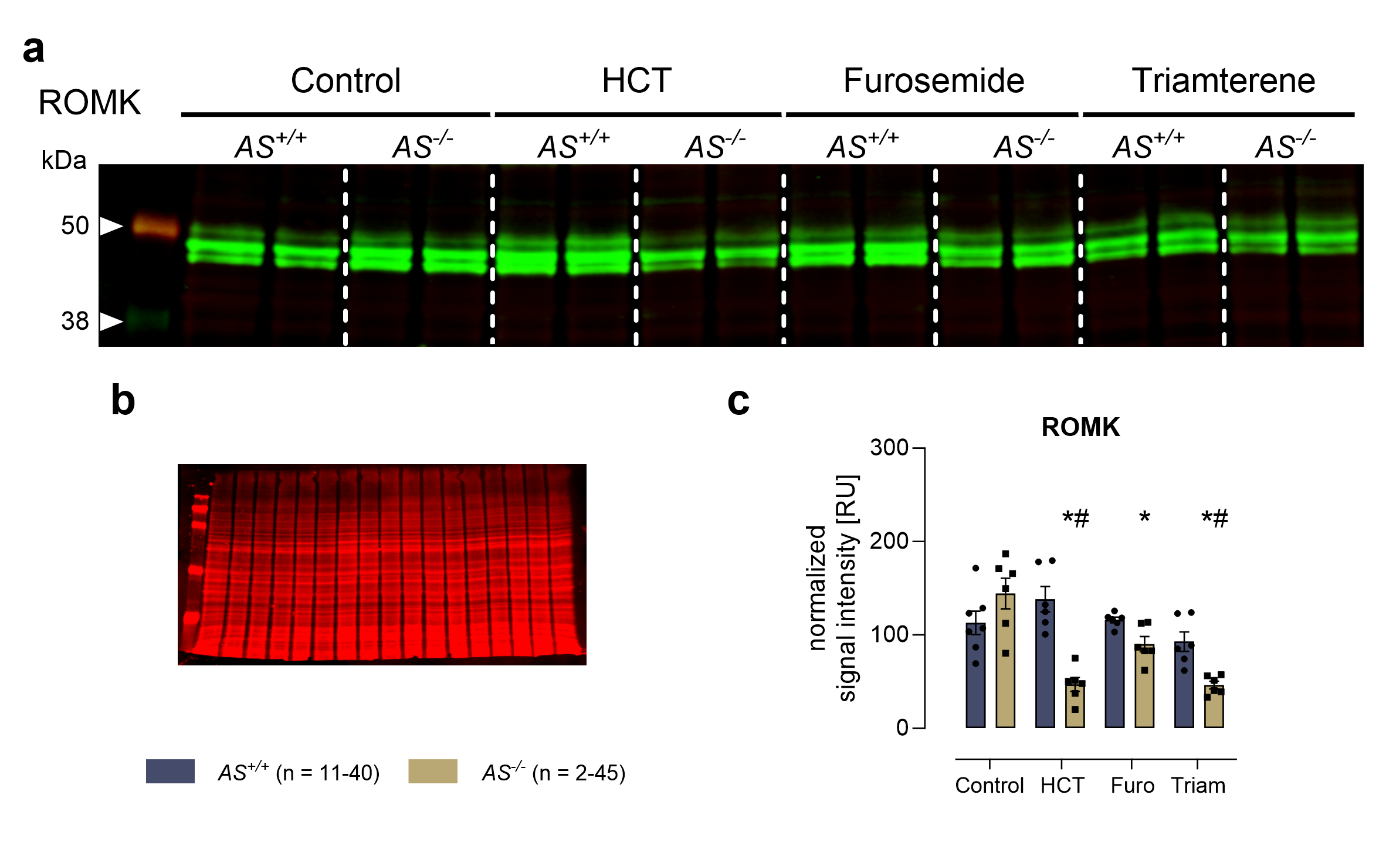


a Representative Western blot showing the expression of ROMK in a plasma membrane preparation of kidney lysates before (control) and after diuretic treatment

b Total protein stain as a loading control

c Densitometry of the obtained bands (between 45 and 50kDa) normalized for total protein content of each lane

# p<0.05 compared to vehicle treatment, * p<0.05 between genotypes

**Supplemental Figure 7: mRNA expression of the sodium transporters and their regulators under control conditions and diuretic treatment in *AS^+/+^* and *AS^-/-^* mice**


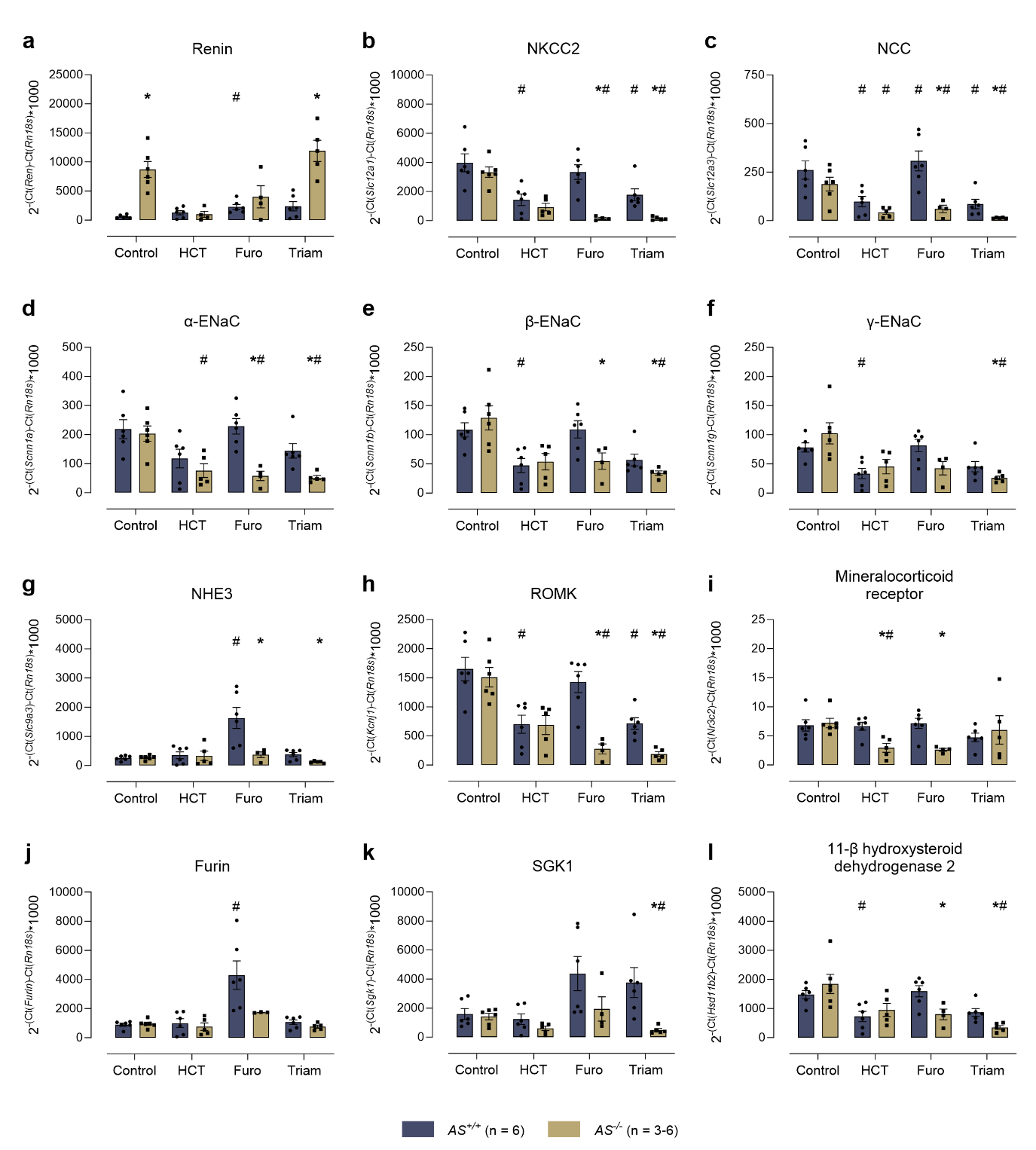


Relative mRNA expression of renin (*Ren*) (a), NKCC2 (*Slc12a1*) (b), NCC (*Slc12a3*) (c), ENaC subunits - (*Scnn1a*) (d), β-(*Scnn1b*) (e), and γ-ENaC (*Scnn1g*) (f), NHE3 (*Slc9a3*) (g), ROMK (*Kcnj1*) (h), mineralocorticoid receptor (*Nr3c2*) (i), furin (*Furin*) (j), SG1 (*Sgk1*) (k) and 11-β hydroxysteroid dehydrogenase 2 (*Hsd11b2*) (l) under control treatment and after diuretic treatment

# p<0.05 compared to vehicle treatment, * p<0.05 between genotypes

**Supplemental Table 1. Used primers.**

| **gene** | **sense/forward**  **5’🡪3’ orientation** | **antisense/reverse**  **5’🡪3’ orientation** | **amplicon** | **reference** |
| --- | --- | --- | --- | --- |
| *Slc12a3* | CTTCGGCCACTGGCATTCTG | GATGGCAAGGTAGGAGATGG | 122 bp | ^26^ |
| *Slc12a1* | GAGATTGGCGTGGTCATAGTCAGAA | TGCTGCTGATGTTGCCGTCTTT | 229 bp | ^27^ |
| *Slc9a3* | AAGCTGTACAAGAGGGAGCG | TGATGGTGTAGTTGTGTGCC | 97 bp | ^27^ |
| *Sgk1* | tgtcttggggctgtcctgtatg | gcttctgctgcttccttcacac | 407 bp | ^28,29^ |
| *Nr3c2* | GAAGAGCCCCTCTGTTTGCAG | TCCTTGAGTGATGGGACTGTG | 130 bp | ^30^ |
| *Kcnj1* | CATCATTGTGAGGCTTAAGATTCATT | GCTGTTCAGTTTTGTCTGTGCAA | 167 bp | ^31^ |
| *Scnn1a* | GGTGCACGGTCAGGATGAG | TAGTTGCCTCCGAGGCTGTC | 117 bp | ^41^ |
| *Scnn1b* | TCCTAGCTTGCCTGTTTGGAA | CAGTTGCCATAATCAGGGTAGAAGAT | 79 bp | ^32^ |
| *Scnn1g* | GCAAGCAATCCTGCAGCTTT | CCCAGGTGAGAACATTCAGCA | 102 bp | ^33^ |
| *Ren* | GCCTCAGCAAGACTGATTCC | CCTGGCTACAGCTCACAACA | 201 bp | ^34,35^ |
| *Hsd11b2* | AACCTCTGGGAGAAACGCAAG | GGCATCTACAACTGGGCTAAGG | 153 bp | ^36^ |
| *Furin* | CCA CAT GAC TAC TCT GCT GAT GG | CGA GAG TGA ACT TGG TCA GCG T | 148 bp | ^37^ |
| *Gapdh* | AACGACCCCTTCATTGAC | TCCACGACATACTCAGCAC | 191 bp | ^38^ |
| *Rps13* | CCCAGGTCCGTTTTGTGACT | GTGCTTTCGGACAGCAACAG | 122 bp | ^39^ |
| *Rn18s* | CCCTGCCCTTTGTACACACC | CGATCCGAGGGCCTCACTA | 67 bp | ^36^ |
